# Demographic Differences in Health Personality

**DOI:** 10.1093/geroni/igaa057.2164

**Published:** 2020-12-16

**Authors:** Nicholas Cone

**Affiliations:** Iowa State University, Ames, Iowa, United States

## Abstract

The purpose of this study was to explore demographic differences in health personality. Data consisted of 3,907 participants, 65 years and older. Multivariate analysis of variance with post-hoc testing revealed that women had higher health neuroticism scores than men, but men had higher health extraversion scores than women. Those married reported higher health agreeableness than those not married and young-old participants had higher health extraversion and health openness compared to other age groups. Regional differences included Midwest participants reporting higher health openness but lower health conscientiousness scores when compared to participants from other regions. There were also significant interactions. For example, individuals from geographic areas with predominately White Midwest residents were significantly higher on health neuroticism when compared to Northwest, South, and West regions. The results are helpful for healthcare providers who can tailor intervention approaches to specific populations.

